# Effects of ilaprazole on the steady-state pharmacodynamics of clopidogrel in healthy volunteers: An open-label randomized crossover study

**DOI:** 10.3389/fphar.2022.952804

**Published:** 2022-09-08

**Authors:** Zekang Ye, Pengsheng Chen, Chuchu Tan, Xiaoxuan Gong, Ran Li, Zhou Dong, Inam Ullah, Chen Zhou, Sufeng Zhou, Lijun Xie, Xuemei Hou, Zhihui Han, Qian Gu, Jiazheng Ma, Jianzhen Teng, Yingdan Tang, Zhuanxia Zhang, Haitang Hu, Quankun Zhuang, Juan Chen, Bei Zhu, Feng Shao, Chunjian Li

**Affiliations:** ^1^ Department of Cardiology, The First Affiliated Hospital of Nanjing Medical University, Nanjing, Jiangsu, China; ^2^ Department of Cardiology, Xuzhou Central Hospital, Xuzhou, Jiangsu, China; ^3^ Phase I Clinical Trial Unit, The First Affiliated Hospital of Nanjing Medical University, Nanjing, Jiangsu, China; ^4^ Lizhu Medical Research Institute, Lizhu Group, Zhuhai, Guangdong, China; ^5^ Department of Biostatistics, School of Public Health, Nanjing Medical University, Nanjing, Jiangsu, China; ^6^ Department of Clinical Pharmacology, School of Pharmacy College, Nanjing Medical University, Nanjing, China

**Keywords:** clopidogrel, ilaprazole, drug-drug interaction, maximal platelet aggregation, platelet reactivity index

## Abstract

**Background:** Previous studies have suggested that proton pump inhibitors could impair the antiplatelet effect of clopidogrel. It is uncertain whether ilaprazole affects the antiplatelet effect of clopidogrel. This study aimed to determine the drug-drug interaction between ilaprazole and clopidogrel.

**Methods:** A randomized crossover trial of 40 healthy subjects was performed. Clopidogrel was administered alone or in combination with ilaprazole for 7 days. The maximal platelet aggregation (MPA) to 5 μmol/L adenosine diphosphate was measured by light transmission aggregometry and the platelet reactivity index (PRI) was determined by vasodilator-stimulated phosphoprotein P2Y_12_ assay. High on-treatment platelet reactivity (HOPR) was defined as a MPA of >40%. The inhibition of platelet aggregation (IPA) and PRI in the two phases were compared between two regimens after the last dosing.

**Results:** IPA was comparable between the two regimens at 0, 10 and 24 h (*p* > 0.05), but higher at 4 h in the clopidogrel alone regimen compared with that in the combined treatment regimen (75.66 ± 18.44% vs. 70.18 ± 17.67%, *p* = 0.031). The inhibition of PRI was comparable between the two regimens at 0 and 24 h. There were no significant differences in the area under the time-IPA% curve (AUC) or the incidence of HOPR at all time-points between the two regimens.

**Conclusion:** In healthy subjects, ilaprazole has limited effect on the pharmacodynamics of clopidogrel and it may not be clinically relevant.

**Clinical Trial Registration**: [www.chictr.org.cn], identifier [ChiCTR2000031482].

## Introduction

The introduction of clopidogrel was a milestone in the development of antiplatelet therapy. Dual antiplatelet therapy with clopidogrel and aspirin is associated with a significant reduction of adverse cardiovascular events in patients with coronary atherosclerosis disease, especially in those undergoing percutaneous coronary intervention (Bhatt et al., 2006). Clopidogrel is a prodrug that needs to be metabolized through the cytochrome P450 (CYP450) system to exert antiplatelet activity. CYP450 family 2 subfamily C member 19 genotypes (CYP2C19) contributes predominantly to the bioactivation of clopidogrel and consequently affects its therapeutic response (Kazui et al., 2010; Cattaneo, 2012).

Proton pump inhibitors (PPIs) have been adopted to prevent and treat gastrointestinal bleeding in patients on dual antiplatelet therapy with clopidogrel and aspirin (Levine et al., 2016). However, PPIs, such as omeprazole and esomeprazole, are mainly metabolized *via* CYP2C19 and CYP3A4 isoenzymes, which competitively affect the metabolism of clopidogrel (Li et al., 2004). Omeprazole can cause a 40% reduction of the clopi-H4 (the active clopidogrel metabolite) and consequently a significantly decreased antiplatelet effect while administered in combination of clopidogrel (Angiolillo et al., 2011). The U.S. Food and Drug Administration has issued warnings to concomitant use of omeprazole or esomeprazole with clopidogrel due to the drug-drug interaction (FDA, 2011; Scott et al., 2013).

Ilaprazole, the latest generation of benzimidazole PPI, is non-inferior to the traditional PPIs in inhibiting gastric acid secretion (Wang et al., 2019; Fan et al., 2019; Wang et al., 2011). The *in vitro* microsome tests have shown that ilaprazole is mainly metabolized by non-enzymatic degradation and partially by CYP3A4, but hardly by CYP2C19, which is significantly different from the current PPIs (Wang et al., 2011; Cho et al., 2012; Seo et al., 2012; Pu et al., 2018). It is unknown yet whether ilaprazole could interfere with the pharmacodynamics of clopidogrel. This study was designed to determine the impact of ilaprazole on the antiplatelet effect of clopidogrel in healthy volunteers.

## Methods

### Study design

This is an open-label randomized crossover study to assess the impact of ilaprazole on the antiplatelet effect of clopidogrel in healthy volunteers. Subjects were enrolled in the First Affiliated Hospital of Nanjing Medical University from 3 March 2021, to 5 June 2021. This study was registered at www.chi www.chictr.org.cn (Unique Identifier: ChiCTR2000031482), which complied with the Declaration of Helsinki (64th, 2013) and was approved by the Ethics Committee of the First Affiliated Hospital of Nanjing Medical University (Approval Number: 2020-MD-030). All participants signed the consent form.

### Study subjects

Healthy volunteers were screened according to the inclusion and exclusion criteria. The inclusion criteria were as follows: 1) subjects aged between 18 and 55 years; 2) subjects weighted ≥50 kg in males and ≥45 kg in females, with body mass index between 19 and 26 kg/m^2^; 3) subjects in healthy status assessed by medical history, laboratory test, electrocardiogram, and physical exam (Supplementary Table S1). The exclusion criteria were as follows: 1) allergic to the study drug; 2) abusing drug within 12 months or using drugs within the last 2 weeks; 3) intake of caffeine or xanthine for 48 h; 4) frequent smoking or drinking alcohol for 3 months; 5) pregnant and lactating women, or subjects who plan to give birth within 6 months; 6) with dysphagia or any gastrointestinal diseases; 7) participating in clinical studies within 3 months before screening; 8) with other conditions that made them unsuitable to be recruited at the discretion of the investigators. Subjects were required to avoid caffeine, alcohol, smoking, heavy exercise or any diet that could interfere with study drugs during the study.

### Trial test

We first enrolled 4 subjects for the trial test (Supplementary Figure S1), which was to evaluate the feasibility of the regimens and collect the preliminary data to calculate the sample size for the formal study. Four eligible subjects were recruited to receive clopidogrel 75 mg once daily for 7 days, and then clopidogrel 75 mg in combination with ilaprazole 10 mg once daily for 7 days (first Regimen A, then Regimen B; *n* = 2), or vice versa (first Regimen B, then Regimen A; *n* = 2), with a 10-day interval between the two regimens. Patients was allocated in different regimens as shown in Supplementary Figure S1.

### Study regimens and drugs

**FIGURE 1:**
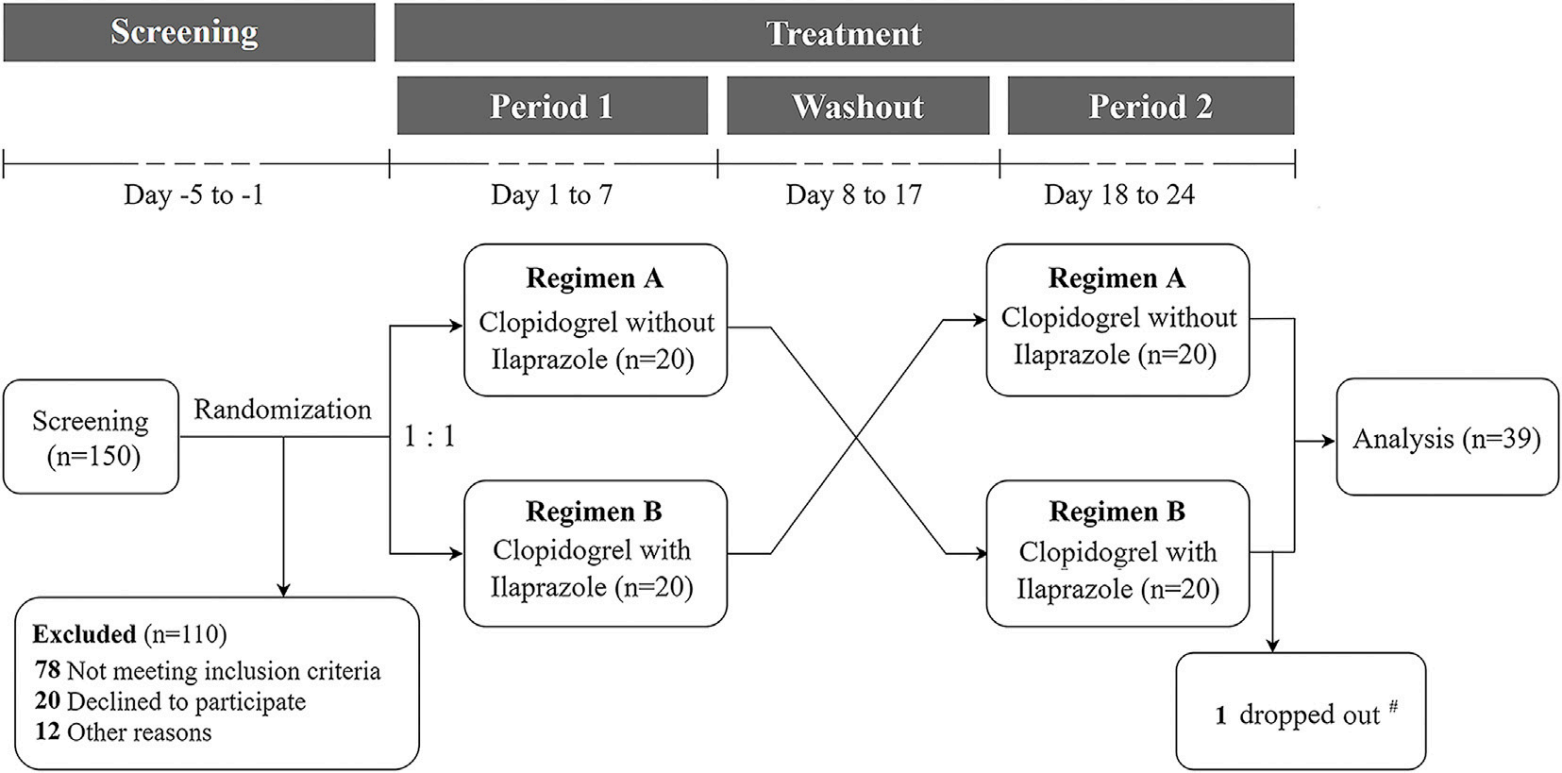
Flow diagram of the formal study. 150 subjects were screened and 40 were randomized in a 1:1 ratio into two protocols to receive clopidogrel 75 mg once daily for 7 days, and then clopidogrel 75 mg in combination with ilaprazole 10 mg once daily for 7 days (first Regimen A, then Regimen B; *n* = 20), or vice versa (first Regimen B, then Regimen A; *n* = 20), with a 10-day interval between the two regimens. # One subject dropped out on Day 18 after the first dosing of Regimen B.

The SAS software, version 9.4 (SAS Institute Inc., Cary, NC, United States) was used to program the randomization algorithm based on the blocked randomization (block size = 4, two arms) by Shanghai Zenith Data Technology Co., Ltd. After screening, 40 subjects were enrolled from 150 healthy volunteers, and randomized in a 1:1 ratio into two groups of AB and BA according to the cross-over design and the predetermined computer-generated random sequence (Figure 1). The drug doses and dosing regimens in the formal study were the same as the trial test. All participants were hospitalized in the clinical trial ward during the medication period (Figure 1).

Clopidogrel (PLAVIX^®^, 75 mg one tablet) was purchased from Sanofi Aventis (Paris, France), and ilaprazole (YILIAN^®^, 10 mg one tablet) was provided by Livzon Pharmaceutical Group Inc (Zhuhai, China). During the study period, all the drugs were stored in a dark environment of no more than 25°C under designated surveillance. After at least 10 h of fasting, the study drugs were orally administered with 240 ml of warm water in a sitting position at 8 a.m. It was confirmed that the drug had been properly taken by examining the patient’s oral cavity and drug containers. Subjects were not allowed to take other drugs during the study period except when adverse events happened and proper drugs were needed.

### Blood collection

Venous blood was collected into three 2.7 ml vacutainer tubes (Becton, Dickinson and Company, Franklin Lakes, United States) containing 0.105 M buffered sodium citrate (3.2%) during the trial test as well as the formal study. The samples were collected before dosing (baseline) and at 0, 4, 10, and 24 h after the last dose of the study drug in each phase. Blood samples were transported, avoiding vigorous shaking, for platelet function assays in a thermostat (20–25°C).

Baseline venous blood was also collected for genotyping into a 2 ml EDTA tube which was frozen at -80 ± 10°C for further analysis.

### Light transmission aggregation

Platelet aggregation tests were performed by two investigators using a Chrono-log Model 700 aggregometer (Chrono-log Corporation, Havertown, PA). Each light transmission aggregation test was completed within 3 h of blood collection. Platelet-rich plasma (PRP) and platelet-poor plasma (PPP) were prepared shortly after blood collection by spinning the sample at 200 g for 5 min in the centrifuge machine. The PRP was carefully removed, and the remaining blood was centrifuged at 2,465 g for 10 min to obtain PPP. The centrifuge temperature was maintained at 22°C. Platelet counts were adjusted by adding PPP to the PRP to achieve a count of 250 × 10^9^/L. Then, 500 μL adjusted PRP was transferred into a test tube, and a 500 μL PPP was set as a control. ADP (with a final concentration of 5 μmol/L) was used as an agonist to induce platelet aggregation. The maximal platelet aggregation (MPA) rates were recorded within 8 min (Pi et al., 2019), and the inhibition of platelet aggregation (IPA) was calculated as follows: $IPA\left(\%\right)=\frac{MPA_{\left(baseline\right)}-MPA_{\left(treatment\right)}}{MPA_{\left(baseline\right)}}\times 100$ . The IPA after the last dosing of the Regimen A or B was defined as primary endpoint of this study. High on-treatment platelet reactivity (HOPR) was defined as MPA >40% (Gurbel et al., 2009).

### VASP P2Y_12_ assay

The vasodilator-stimulated phosphoprotein (VASP) P2Y_12_ assay (Biocytex, Marseille, France) was performed using sodium citrate anticoagulated whole blood as per manufacturer instructions and was stored overnight at 25°C. The analysis was finished within 24 h of blood collection. The blood samples were incubated with PGE1 alone or with PGE1 and ADP simultaneously. After cellular permeabilization, VASP was labeled by indirect no-wash immunofluorescence using a specific monoclonal antibody (clone 16C2). Platelets were identified by flow cytometry in a FACSCalibur flow cytometer (Becton Dickinson), and the level of VASP-Ser239P was simultaneously determined by 16C2-FITC mean fluorescence intensity (MFI). The following formula was applied to calculate the platelet reactivity index (PRI) using corrected MFI (MFIc): $PRI\left(\%\right)=\frac{MFIc_{\left(PGE1\right)}-MFIc_{\left(PGE1+ADP\right)}}{MFIc_{\left(PGE1\right)}}\times 100$ . The percent inhibition of PRI was calculated using the following formula to adjust the possible differences at baseline between the two phases: $PRI_{inhibition}\left(\%\right)=\frac{PRI_{\left(baseline\right)}-PRI_{\left(treatment\right)}}{PRI_{\left(baseline\right)}}\times 100$ .

### Genotype analysis

To compare the pharmacodynamic differences in different CYP2C19 genotypes when clopidogrel was co-administered alone or with ilaprazole, blood samples were transferred through the cold chain at -80 ± 10°C to Suzhou Hongxun Biotechnologies Co., LTD. The CYP2C19*2 (681, G > A) and CYP2C19*3 (636, G > A) were genotyped using Sanger sequencing *via* PCR amplification on an ABI 3730xl DNA Analyzer (Applied Biosystems) (Wang et al., 2021). Subjects with different CYP2C19 gene polymorphisms were defined as extensive metabolizers (EMs, CYP2C19*1/*1), intermediate metabolizers (IMs, CYP2C19*1/*2 and *1/*3) and poor metabolizers (PMs, CYP2C19*2/*2, *2/*3 and *3/*3) (Zhang et al., 2020).

### Statistical analysis

Referring to the similar research on drug-drug interaction between clopidogrel and other PPIs (Funck-Brentano et al., 2013; Frelinger et al., 2012), the inter-group variation of IPA between regimens of clopidogrel alone and the combination of clopidogrel and omeprazole was about 15%. We presumed that the difference of IPA between the two regimens was 8.6% based on the results from the trial test. A total sample size of 36 participants (18 per group) was calculated to detect the prespecified effect size at a two-sided 0.05 significance level and a power of 90%. The sample size was adjusted for an anticipated 10% drop-out rate yielding a final sample size of 40 participants.

Statistical analysis was performed using SAS software, version 9.4 (SAS Institute Inc., Cary, NC, United States). Continuous variables were expressed as mean ± standard deviation (SD) or median with interquartile range (IQR) when data did not follow a normal distribution. Categorical variables were presented as frequencies and percentages. Subjects who participated in the formal trial were included in the final analysis according to the per-protocol set (PPS).

A generalized linear mixed-model approach was used to compare the pharmacodynamic indexes between the two treatment regimens. The estimated treatment difference, 95% confidential interval (CI) and *p*-value were adjusted with sequence, phase, and treatment as fixed factors, and subjects within the sequence as a random factor.

The sensitivity analysis was performed by adding the gender and CYP2C19 metabolizer into the generalized linear mixed-model. To evaluate the influence of gender and CYP2C19 metabolizer on the interaction between clopidogrel and ilaprazole, the interactions of gender, CYP2C19 metabolizer and treatment regimen were included in the generalized linear mixed-model.

Multilevel logistic regression models were used to compare the incidence of HOPR between the two treatment regimens. Pre-planned subgroup analysis was used to compare the effects of ilaprazole on clopidogrel among different CYP2C19 genotypes.

A two-sided *p* value of <0.05 was considered statistically significant for all analyses.

## Results

### Study subjects

A total of 150 volunteers were consecutively screened, of whom 40 subjects who met the inclusion and exclusion criteria were included and randomized into the two study regimens. One subject dropped out the study on Day 18 after the first dosing of Regimen B. As a result, 39 subjects completed the study and were included in the final analyses (Figure 1). The demographic characteristics of the included subjects are shown in Table 1.

**TABLE 1:** Demographic characteristics by sequence.

Characteristics	AB (*n* = 20)	BA (*n* = 20)
Male, *n* (%)	16 (80.0)	18 (90.0)
Asian Race, *n* (%)	20 (100.0)	20 (100.0)
Age (years)	26.7 ± 4.9	31.0 ± 6.8
Height (cm)	170.1 ± 10.1	168.8 ± 6.5
Weight (kg)	65.9 ± 9.6	62.9 ± 6.2
BMI (kg/m^2^)	22.7 ± 2.0	22.0 ± 1.4

Values are presented as *n* (%) or mean ± SD., Regimen A: clopidogrel 75 mg once daily; regimen B: clopidogrel 75 mg with ilaprazole 10 mg once daily. Abbreviation: BMI, body mass index.

### Light transmission aggregation

Baseline MPA was comparable between the two regimens of clopidogrel alone and clopidogrel plus ilaprazole (59.38 ± 21.69% vs. 64.97 ± 20.82%, *p* = 0.085). After taking the study drugs for 7 days, the MPA was significantly lower in the clopidogrel alone regimen compared with clopidogrel plus ilaprazole regimen at 0 h (19.21 ± 11.30% vs. 23.67 ± 12.99%, *p* = 0.001), 4 h (14.36 ± 10.22% vs. 18.49 ± 10.08%, *p* < 0.001), 10 h (16.51 ± 11.26% vs. 19.62 ± 10.83%, *p* < 0.001) and 24 h (19.28 ± 12.47% vs. 22.62 ± 11.73%, *p* < 0.001) (Supplementary Table S2).

**FIGURE 2:**
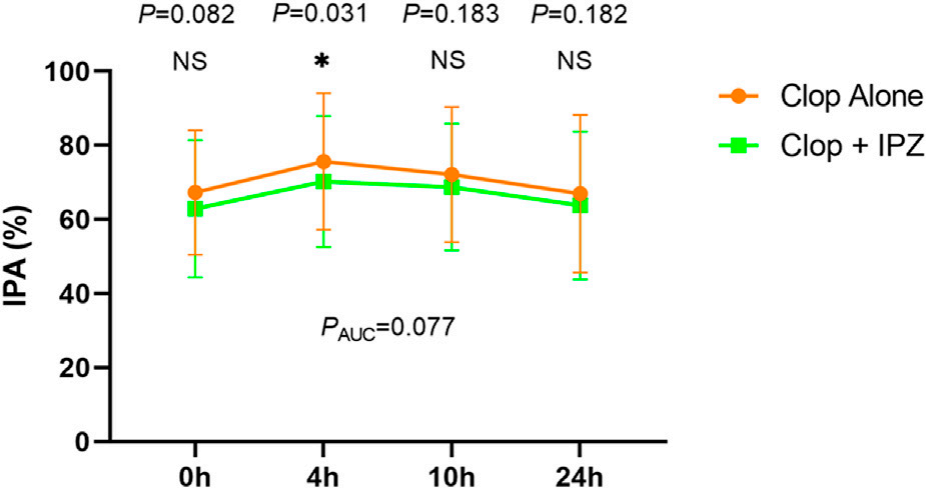
IPA-time curve after 7-day treatment of the two regimens. Data are expressed as the mean ± SD (*n* = 39). IPA = inhibition of platelet aggregation; Clop = clopidogrel; IPZ = ilaprazole. NS = not significant. AUC = the area under the time-IPA% curve. * represents *p* < 0.05.

The IPA was significantly higher at 4 h after 7-day administration of clopidogrel compared to coadministration of clopidogrel and ilaprazole (75.66 ± 18.44% vs. 70.18 ± 17.67%, *p* = 0.031). However, the IPA levels were comparable at 0 h (67.28 ± 16.80% vs. 62.88 ± 18.51%, *p* = 0.082), 10 h (72.12 ± 18.27% vs. 68.74 ± 17.14%, *p* = 0.183) and 24 h (66.96 ± 12.23% vs. 63.76 ± 19.97%, *p* = 0.181) between the two regimens (Figure 2; Supplementary Table S2).

There was no significant difference in the area under the time-IPA% curve (AUC) between the two regimens (1702.78 ± 430.75% h vs. 1,610.42 ± 410.29% h, *P*_AUC_ = 0.077) (Figure 2). Besides, the incidences of HOPR were not significantly different between the two regimens at 0, 4, 10 and 24 h after 7-day administration of the study drugs (Table 2).

**TABLE 2:** HOPR status on basis of MPA after the regimen of clopidogrel alone compared with coadministration of clopidogrel with ilaprazole.

Time (h)	*n*	Clop alone	Clop + IPZ	*p*-value
0	39	2 (5.1%)	3 (7.7%)	0.998
4	39	1 (2.6%)	0	0.999
10	39	2 (5.1%)	3 (7.7%)	0.998
24	39	3 (7.7%)	3 (7.7%)	0.999

Abbreviations: HOPR, high on-treatment platelet reactivity; MPA, maximal platelet aggregation; Clop: clopidogrel; IPZ: ilaprazole.

### VASP P2Y_12_ assay

Before administration of the study drugs, baseline PRI was not significantly different between the clopidogrel alone and the coadministration regimens (91.16 ± 3.55% vs. 90.68 ± 3.79%, *p* = 0.527). The PRI was significantly lower in the regimen of clopidogrel alone compared to coadministration of clopidogrel and ilaprazole at 4 h (54.69 ± 22.21% vs. 59.60 ± 21.15%, *p* = 0.003) and 10 h (56.25 ± 19.95% vs. 61.38 ± 18.76%, *p* = 0.006). However, PRI was not statistically different between the two regimens at 0 h (63.22 ± 18.17% vs. 65.52 ± 19.58%, *p* = 0.527) and 24 h (60.33 ± 17.85% vs. 62.87 ± 17.54%, *p* = 0.076) (Table 3).

**TABLE 3:** PRI and PRI_Inhibition_ by VASP P2Y_12_ assay in subjects under clopidogrel treatment with or without ilaprazole.

Time (h)	Clop alone	Clop + IPZ	Difference	95% CI	*p*-value
PRI (%)					
Baseline	91.16 ± 3.55	90.68 ± 3.79	0.49	(1.01, −1.99)	0.527
0	63.22 ± 18.17	65.52 ± 19.58	−2.34	(−5.17, 0.49)	0.114
4	54.69 ± 22.21	59.60 ± 21.15	−4.94	(−7.97, −1.91)	0.003
10	56.25 ± 19.95	61.38 ± 18.76	−5.20	(−8.69, −1.71)	0.006
24	60.33 ± 17.85	62.87 ± 17.54	−2.51	(−5.21, 0.18)	0.076
PRI_Inhibition_ (%)					
0	30.81 ± 19.11	27.72 ± 21.57	3.14	(−0.27, 6.54)	0.080
4	40.20 ± 23.63	34.28 ± 23.29	5.95	(2.35, 9.55)	0.003
10	38.50 ± 21.14	32.35 ± 20.43	6.23	(2.47, 9.99)	0.003
24	33.92 ± 18.95	30.62 ± 19.40	3.27	(−0.06, 6.47)	0.053

Values are presented as mean ± SD. Abbreviations: PRI, platelet reactivity index; VASP, vasodilator-stimulated phosphoprotein; Clop: clopidogrel; IPZ: ilaprazole; CI, confidential interval; PRI_Inhibition_, inhibition of platelet reactivity index.

Further analysis shows that PRI_Inhibition_ was significantly higher after 7-day administration of clopidogrel compared to coadministration of clopidogrel and ilaprazole at 4 h (40.20 ± 23.63% vs. 34.28 ± 23.29%, *p* = 0.003) and 10 h (38.50 ± 21.14% vs. 32.35 ± 20.43%, *p* = 0.003). However, the levels of PRI_Inhibition_ were similar at 0 h (30.81 ± 19.11% vs. 27.72 ± 21.57%, *p* = 0.080) and 24 h (33.92 ± 18.95% vs. 30.62 ± 19.40%, *p* = 0.053) in the two regimens (Table 3).

### CYP2C19 genotyping and subgroup analysis

The CYP2C19 genotype analysis showed that there were 9 (23%) EMs, 22 (56.4%) IMs and 8 (20.5%) PMs in the participants. As the interaction between ilaprazole and clopidogrel was significant at 4 h after medication for both IPA and PRI_Inhibition_ (Supplementary Table S2; Table 3), the pharmacodynamics of clopidogrel at this timepoint were selected for the subgroup analysis by different CYP2C19 genotypes.

**FIGURE 3:**
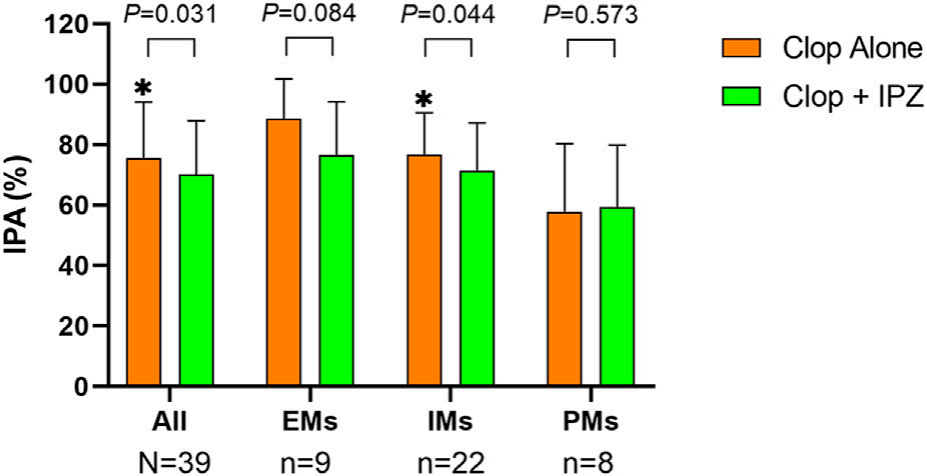
IPA at 4 h after 7-day treatment in different CYP2C19 genotypes. Upper boundaries of boxes represent means; upper whiskers represent standard deviations of IPA. IPA = inhibition of platelet aggregation; Clop = clopidogrel; IPZ = ilaprazole; EMs = extensive metabolizers (CYP2C19*1/*1); IMs = intermediate metabolizers (CYP2C19*1/*2 and *1/*3); PMs = poor metabolizers (CYP2C19*2/*2, *2/*3 and *3/*3). * represents *p* < 0.05.

In IMs, the IPA was significantly higher at 4 h after medication in the clopidogrel alone regimen than that in the combination regimen (76.84 ± 13.66% vs. 71.49 ± 15.63%, *p* = 0.044). However, the IPA were comparable between the two regimens in both EMs (88.58 ± 13.20% vs. 76.53 ± 17.66%, *p* = 0.084) and PMs (57.85 ± 22.49% vs. 59.42 ± 20.42%, *p* = 0.573) (Figure 3; Supplementary Table S3).

PRI_Inhibition_ was significantly higher at 4 h in the regimen of clopidogrel alone compared to coadministration of clopidogrel and ilaprazole in both EMs (62.99 ± 18.58% vs. 52.69 ± 24.93%, *p* = 0.036) and IMs (38.14 ± 20.95% vs. 32.70 ± 21.46%, *p* = 0.037). However, it was comparable between the two regimens (20.20 ± 13.46% vs. 17.91 ± 10.18%, *p* = 0.251) in PMs (Supplementary Table S3).

### Sensitivity analysis

After adjusted for gender and CYP2C19 metabolizer, the difference of IPA at 4 h (*p* = 0.031) as well as the difference of PRI_Inhibition_ at 4 h (*p* = 0.003) and 10 h (*p* = 0.003) between the two regimens kept statistically significant. However, only CYP2C19 metabolizer was significantly related to the IPA at 4 h (*p* = 0.014) and PRI_Inhibition_ at 4 h (*p* = 0.0004) and 10 h (*p* = 0.0006), while gender was not significantly related to either the IPA at 4 h (*p* = 0.402), or PRI_Inhibition_ at 4 h (*p* = 0.598) and 10 h (*p* = 0.934).

### Interaction analysis

After adding gender, CYP2C19 metabolizer, and the interactions between these two factors and treatment regimen in the generalized linear mixed-model, the interactions between gender (*p* = 0.127), metabolizer (*p* = 0.069) and treatment regimen were not statistically significant for IPA at 4 h. Similarly, the interactions between gender, metabolizer and treatment regimen were also not significant for PRI_Inhibition_ at 4 h (*p* = 0.135 for gender and 0.252 for metabolizer) and 10 h (*p* = 0.135 for gender and 0.195 for metabolizer).

## Discussion

To the best of our knowledge, this is the first prospective study investigating the drug-drug interaction between clopidogrel and ilaprazole. We found that, after being treated for 7 days, the IPAs were comparable between the two regimens at all time-points of 0, 10 and 24 h, except that at 4 h.

Regarding the study design, the routine recommended doses of both clopidogrel and ilaprazole were adopted. Referring to previous studies (Thebault et al., 1999; Li et al., 2012; Shin et al., 2014), 7-day treatment and 10-day interval were chosen. As this study aimed to investigate the drug-drug interaction of clopidogrel and ilaprazole, healthy volunteers instead of patients were recruited, and the laboratory endpoint of IPA instead of clinical events was set as the primary endpoint.

After baseline adjustment, no statistical difference of IPAs was found between the two regimens at all time-points except that at 4 h. Besides, the areas under the time-IPA% curves were comparable between the two regimens. These results indicated that the interaction between clopidogrel and ilaprazole was limited. Our study revealed that the difference of IPA at 4 h was 5.52%, which was less than the impact of other PPIs reported in previous studies (Angiolillo et al., 2011; Frelinger et al., 2012). Frelinger, A.L. et al. Found that the decrease in IPA was 12.9%, 11.6% respectively at 24 h after 9 days of coadministration of omeprazole or esomeprazole with clopidogrel (Frelinger et al., 2012). Another study by Angiolillo, D.J. et al. reported that the IPA decreased by 7.7% at 2, 4 and 6 h after 5-day coadministration of clopidogrel and pantoprazole compared with clopidogrel alone (Angiolillo et al., 2011).

The MPAs were statistically higher at all time-points in the coadministration regimen compared with those in the clopidogrel alone regimen. However, a difference of MPA>10% was suggested to be the indication of clinically relevant effect in other studies (Angiolillo et al., 2011; Hochholzer et al., 2006). By comparison, none of the MPA difference was beyond this range in our study, which suggested that the differences of MPAs caused by ilaprazole might have no clinical impact.

The differences of PRIs between the two regimens were statistically significant at 4 h [−4.94 (−7.97, −1.91)] and 10 h [−5.20 (−8.69, −1.71)], respectively. However, as the equivalence range of (−15%, 15%) for PRI was recommended previously (Frelinger et al., 2012), our results suggested a limited impact of ilaprazole on the antiplatelet effect of clopidogrel.

It should be noted that HOPR has been regarded as a risk factor of major adverse cardiovascular events in coronary atherosclerosis disease patients (Ying et al., 2021). Our results demonstrated that the incidence of HOPR was not significantly increased in the coadministration regimen at any time-point. Besides, no significant increase of major adverse cardiovascular events was demonstrated when greater degrees of drug-drug interaction existed between other PPIs and clopidogrel (Bhatt et al., 2010; Lin et al., 2012).

Studies have investigated the interactions between multiple PPIs and clopidogrel. Lin SF and Przespolewski et al. found no interactions between lansoprazole, esomeprazole, pantoprazole, rabeprazole and clopidogrel in Asian patients or healthy male participants (Lin et al., 2020; Przespolewski et al., 2018). However, it was found that omeprazole and dexlansoprazole could affect the anti-platelet effect of clopidogrel (Lin et al., 2020; Furtado et al., 2016). Up to date, only one latest study retrospectively investigated the interaction between clopidogrel and ilaprazole in acute stroke patients, which proved that the combination therapy of ilaprazole does not interfere with the metabolism of clopidogrel [Lim et al., 2022]. It could be concluded that no robust interaction between clopidogrel and PPIs was found. Our results add data to a growing body of evidence indicating that the addition of a PPI may have a weak effect on clopidogrel’s antiplatelet properties, which may not be clinically relevant.

Our study proved that the CYP2C19 metabolizers were significantly related to the pharmacodynamics of clopidogrel, which was consistent with the previous study results (Xu et al., 2019). However, the interaction analysis demonstrated that the CYP2C19 metabolizers had no influence on the interaction between ilaprazole and clopidogrel. Additionally, we found that gender had neither significant effect on the pharmacodynamics of clopidogrel nor the interaction between ilaprazole and clopidogrel.

It is well known that about 50% of clopidogrel is absorbed from the intestine after administration. Once delivered to the liver, a number of CYP450 enzymes, including CYP2C19, CYP1A2, CYP2B6, CYP2C9 and CYP3A4, mediate the bioactivation of clopidogrel *via* a two-step process (Jiang et al., 2015). CYP2C19 contributes to the two steps of clopidogrel metabolism by 45%, 20% respectively, in which clopidogrel is metabolized to 2-oxo-clopidogrel and active metabolite (Kazui et al., 2010). Ilaprazole, however, is not metabolized by CYP2C19, but by non-enzymatic sulfoxide and partially oxidized by CYP3A4, which contributes to the second step of clopidogrel metabolism by 40% (Kazui et al., 2010; Pu et al., 2018; Funck-Brentano et al., 2013). Our study showed that ilaprazole caused changes in the pharmacodynamics of clopidogrel in a certain degree at some time-points. However, the underlying mechanism may not be related to CYP2C19. The real mechanism remains to be further clarified.

### Strengths

This was an open-label randomized crossover study, which has been carefully designed to control possible baseline conditions affecting clopidogrel and/or PPI metabolism. During the treatment period, subjects were hospitalized and confined to receive a uniform diet and refrained from factors that might affect or compromise clopidogrel’s efficacy, including caffeine, alcohol, smoking, and strenuous exercise.

### Limitations

First, no other PPIs were set as controls to compare the impacts of ilaprazole and other PPIs on the pharmacodynamics of clopidogrel. Second, our study was confined to young healthy subjects and the results need further validation in patients.

## Conclusion

In healthy subjects, ilaprazole has limited effect on the pharmacodynamics of clopidogrel and it may not be clinically relevant.

## Data Availability

The genotypes presented in the study are available in the Supplementary Table 4. Further inquiries for other data can be directly from the corresponding authors on reasonable request. The original contributions presented in the study are publicly available. This data can be found here: https://www.ncbi.nlm.nih.gov/clinvar/ SCV00256845 and SCV002568452.

## Supplementary material

Supplementary Figure S1 Flow diagram of the trial test. Four eligible subjects were recruited to receive clopidogrel 75 mg once daily for 7 days, and then clopidogrel 75 mg in combination with ilaprazole 10 mg once daily for 7 days (first Regimen A, then Regimen B; *n*=2), or vice versa (first Regimen B, then Regimen A; *n*=2), with a 10-day interval between the two regimens.
