# Supplementary material for: Effects of ilaprazole on the steady-state pharmacodynamics of clopidogrel in healthy volunteers: An open-label randomized crossover study
Source: Front Pharmacol. 2022 Sep 8;13:952804. doi: 10.3389/fphar.2022.952804 (PMC9492925; doi:10.3389/fphar.2022.952804)
Supplement: Supplementary file 1 [file Table1.DOCX]

**Table S1.** Schedule of study procedures

| **Procedure** | **Pre-treatment** | | **Period 1** | **Washout period** | | | | **Period 2** | **Oberservation** | **Discharge** |
| --- | --- | --- | --- | --- | --- | --- | --- | --- | --- | --- |
| **Visit Time** | **Days -5 to -2** | **Day -1** | **Days 1 to 7** | **Day 8** | **Day 9** | **Days 10 to 15** | **Day 17** | **Days 18 to 24** | **Day 25** | **Day 26** |
| Informed consent | × |  |  |  |  |  |  |  |  |  |
| Demographic data | × |  |  |  |  |  |  |  |  |  |
| Medical history | × | ×^4^ |  |  |  |  | × |  |  |  |
| Height and weight | × |  |  |  |  |  |  |  |  |  |
| Genotype test |  | × |  |  |  |  |  |  |  |  |
| Vital signs ^1^ | × | ×^4^ | × | × | × |  | × | × | × | × |
| Physical examination | × |  |  |  |  |  |  |  |  | × |
| Blood routine test | × | ×^4, 5^ |  |  |  |  | × |  |  | × |
| Urine routine test | × | ×^4, 5^ |  |  |  |  | × |  |  | × |
| Blood biochemistry test | × | ×^4, 5^ |  |  |  |  | × |  |  | × |
| Coagulation function test | × | ×^4, 5^ |  |  | × |  | × |  |  | × |
| 12-lead ECG | × | ×^4, 5^ |  |  |  |  | × |  |  | × |
| Serum pregnancy test | × | ×^4^ |  |  |  |  | × |  |  | × |
| HBV, HCV, HIV and TP screening ^2^ | × |  |  |  |  |  |  |  |  |  |
| Urine drug screening | × | ×^4^ |  |  |  |  | × |  |  |  |
| Alcohol breath test | × | ×^4^ |  |  |  |  | × |  |  |  |
| Inclusion/exclusion criteria | × | ×^4^ |  |  |  |  | × |  |  |  |
| Exit criteria |  |  | × | × | × |  |  | × | × | × |
| Admission |  | × |  |  |  |  | × |  |  |  |
| Dosing |  |  | × |  |  |  |  | × |  |  |
| Pharmacodynamic analysis ^3^ |  |  | × | × |  |  |  | × | × |  |
| Concomitant treatment |  |  |  | ×^6^ | | | | | | |
| Adverse events evaluation |  |  |  | ×^6^ | | | | | | |
| Serious adverse events evaluation |  |  |  | ×^6^ | | | | | | |

1. Vital signs: The subjects were required to be examined of blood pressure, pulse and ear temperature after resting for 3 to 5 minutes before medication on the day of admission and discharge. Only blood pressure and pulse were measured 4h and 8h after dosing.
2. HBV screening included the detection of hepatitis B surface antigen (HBsAg), hepatitis B surface antibody (HBsAb), hepatitis B e antigen (HBeAg), hepatitis B e antibody (HBeAb) and hepatitis B core antibody (HBcAb). HCV, HIV and TP screening included the detection of hepatitis C virus antibody (HCV-Ab), human immunodeficiency virus antibody (HIV-Ab) and treponema pallidum antibody (TP-Ab).
3. Blood samples were collected at 0-hour pre-dose on Day 1 and Day 18 to measure the baseline values of platelet maximum aggregation (MPA) and platelet response index (PRI). Blood samples were collected at 0-hour pre-dose and 4, 10, 24 hours post-dose on Day 7 and Day 24 to measure MPA and PRI.
4. Subjects who had been screened in Day -1 were exempted from the inspection when they checked in on day -1.
5. The blood routine test, urine routine test, blood biochemistry test, coagulation function test, and electrocardiogram were detected again at the time of admission on day -1 when the interval between screening and dosing was ≥7 days.
6. Any combined treatment or adverse events that occurred after dosing during the study were recorded in time and followed up till they recovered to a normal status judged by the researcher.
