# Supplementary figures and images for: Effects of ilaprazole on the steady-state pharmacodynamics of clopidogrel in healthy volunteers: An open-label randomized crossover study
Source: Front Pharmacol. 2022 Sep 8;13:952804. doi: 10.3389/fphar.2022.952804 (PMC9492925; doi:10.3389/fphar.2022.952804)

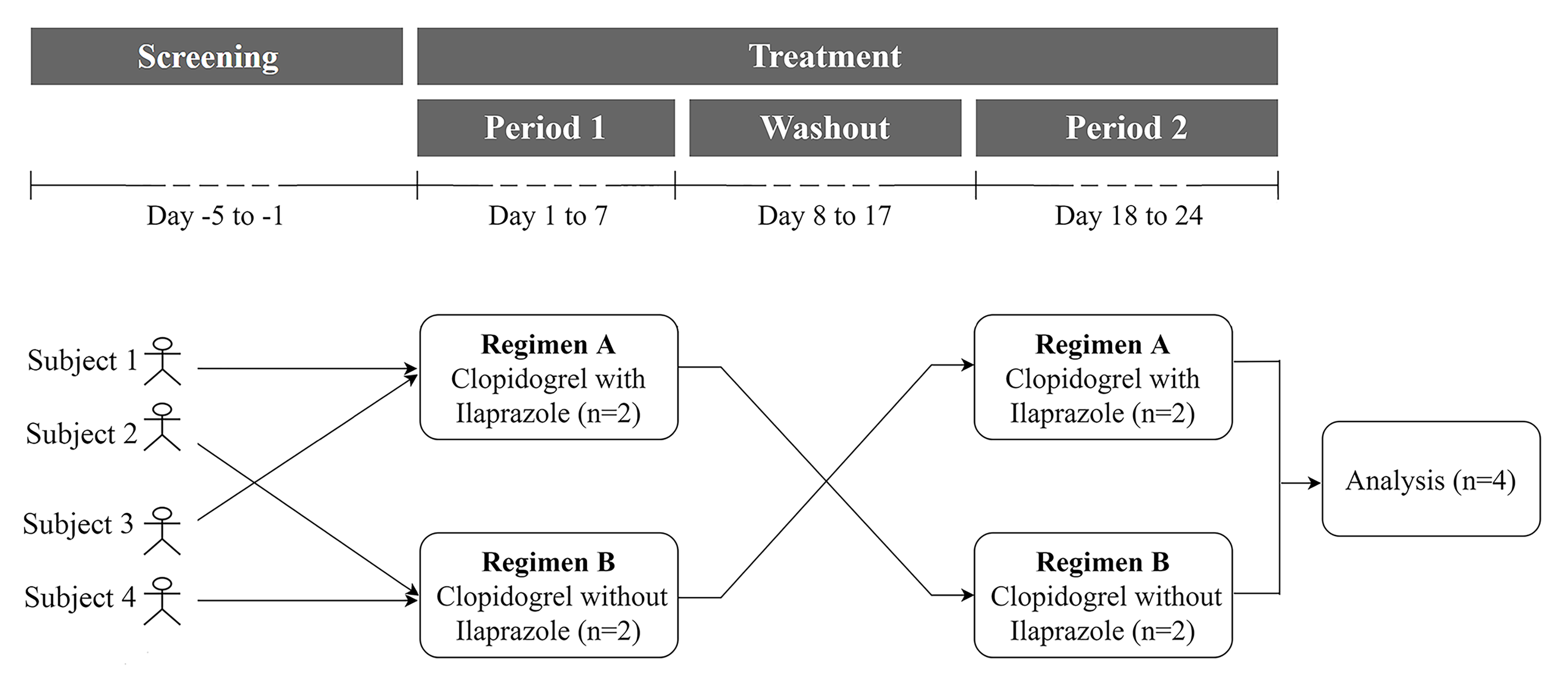

Supplement: Supplementary file 2 [file Image1.TIF]
