# Supplementary material for: Effects of ilaprazole on the steady-state pharmacodynamics of clopidogrel in healthy volunteers: An open-label randomized crossover study
Source: Front Pharmacol. 2022 Sep 8;13:952804. doi: 10.3389/fphar.2022.952804 (PMC9492925; doi:10.3389/fphar.2022.952804)
Supplement: Supplementary file 3 [file Table2.DOCX]

**Table S2.** Platelet aggregation measured by LTA in subjects under the treatment of clopidogrel alone or with ilaprazole

| Time (h) | | Clop alone | | Clop + IPZ | | Difference | 95% CI | *P*-value |
| --- | --- | --- | --- | --- | --- | --- | --- | --- |
| MPA (%) | |  |  |  |  |  |  |  |
| Baseline | | 59.38 ± 21.69 | | 64.97 ± 20.82 | | -5.73 | (-12.08, 0.61) | 0.085 |
| 0 | | 19.21 ± 11.30 | | 23.67 ± 12.99 | | -4.48 | (-7.01, -1.95) | 0.001 |
| 4 | | 14.36 ± 10.22 | | 18.49 ± 10.08 | | -4.19 | (-6.29, -2.10) | <0.001 |
| 10 | | 16.51 ± 11.26 | | 19.62 ± 10.83 | | -3.13 | (-4.74, -1.52) | <0.001 |
| 24 | | 19.28 ± 12.47 | | 22.62 ± 11.73 | | -3.40 | (-5.05, -1.74) | <0.001 |
| IPA (%) |  | | | | | | | |
| 0 | | 67.28 ± 16.80 | | 62.88 ± 18.51 | | 4.32 | (-0.40, 9.04) | 0.082 |
| 4 | | 75.66 ± 18.44 | | 70.18 ± 17.67 | | 5.52 | (0.72, 10.33) | 0.031 |
| 10 | | 72.12 ± 18.27 | | 68.74 ± 17.14 | | 3.36 | (-1.48, 8.20) | 0.183 |
| 24 | | 66.96 ± 12.23 | | 63.76 ± 19.97 | | 3.21 | (-1.40, 7.83) | 0.181 |

Values are presented as mean ± SD. Abbreviations: LTA *=* light transmission aggregometry; Clop = clopidogrel; IPZ = ilaprazole; CI *=* confidential interval; MPA *=* maximal platelet aggregation; IPA *=* inhibition of platelet aggregation.
