# Supplementary material for: Effects of ilaprazole on the steady-state pharmacodynamics of clopidogrel in healthy volunteers: An open-label randomized crossover study
Source: Front Pharmacol. 2022 Sep 8;13:952804. doi: 10.3389/fphar.2022.952804 (PMC9492925; doi:10.3389/fphar.2022.952804)
Supplement: Supplementary file 4 [file Table3.DOCX]

**Table S3.** PRI_inhibition_ and IPA at 4h in subjects under the treatment of 7-day clopidogrel alone or with ilaprazole in CYP2C19 genotypes subgroup

| Genotype | Clop alone | | Clop + IPZ | | Difference | 95% CI | *P*-value |
| --- | --- | --- | --- | --- | --- | --- | --- |
| IPA (%) |  |  |  |  |  |  |  |
| EMs | 88.58 ± 13.20 | | 76.53 ± 17.66 | | -10.90 | (-21.34, 0.46) | 0.084 |
| IMs | 76.84 ± 13.66 | | 71.49 ± 15.63 | | -5.56 | (-10.60, -0.52) | 0.044 |
| PMs | 57.85 ± 22.49 | | 59.46 ± 20.42 | | -6.95 | (-29.41,15.51) | 0.573 |
| PRI_Inhibition_ (%) | | | | | | | |
| EMs | 62.99 ± 18.58 | | 52.68 ± 24.93 | | -11.63 | (-20.28, -2.98) | 0.036 |
| IMs | 38.14 ± 20.95 | | 32.70 ± 21.46 | | -5.36 | (-10.04, -0.67) | 0.037 |
| PMs | 20.20 ± 13.46 | | 17.91 ± 10.18 | | -7.38 | ( -18.55, 3.79) | 0.251 |

Values are presented as mean ± SD. Abbreviations: Clop = clopidogrel; IPZ = ilaprazole; EMs = extensive metabolizers; IMs = intermediate metabolizers; PMs = poor metabolizers; CI *=* confidential interval; IPA *=* inhibition of platelet aggregation; PRI_inhibition_ *=* the percent inhibition of PRI.
